# UBE3A: An E3 Ubiquitin Ligase With Genome-Wide Impact in Neurodevelopmental Disease

**DOI:** 10.3389/fnmol.2018.00476

**Published:** 2019-01-04

**Authors:** Simon Jesse Lopez, David J. Segal, Janine M. LaSalle

**Affiliations:** ^1^Department of Medical Immunology and Microbiology, University of California, Davis, Davis, CA, United States; ^2^Genome Center, University of California, Davis, Davis, CA, United States; ^3^MIND Institute, University of California, Davis, Davis, CA, United States; ^4^Integrative Genetics and Genomics, University of California, Davis, Davis, CA, United States; ^5^Department of Biochemistry and Molecular Medicine, University of California, Davis, Davis, CA, United States

**Keywords:** neurodevelopment, parental imprinting, human genetics and genomics, synapse, Angelman syndrome, autism (ASD)

## Abstract

UBE3A is an E3 ubiquitin ligase encoded by an imprinted gene whose maternal deletion or duplication leads to distinct neurodevelopment disorders Angelman and Dup15q syndromes. Despite the known genetic basis of disease, how changes in copy number of a ubiquitin ligase gene can have widespread impact in early brain development is poorly understood. Previous studies have identified a wide array of UBE3A functions, interaction partners, and ubiquitin targets, but no central pathway fully explains its critical role in neurodevelopment. Here, we review recent UBE3A studies that have begun to investigate mechanistic, cellular pathways and the genome-wide impacts of alterations in UBE3A expression levels to gain broader insight into how UBE3A affects the developing brain. These studies have revealed that UBE3A is a multifunctional protein with important nuclear and cytoplasmic regulatory functions that impact proteasome function, Wnt signaling, circadian rhythms, imprinted gene networks, and chromatin. Synaptic functions of UBE3A interact with light exposures and mTOR signaling and are most critical in GABAergic neurons. Understanding the genome-wide influences of UBE3A will help uncover its role in early brain development and ultimately lead to identification of key therapeutic targets for UBE3A-related neurodevelopmental disorders.

## Introduction to UBE3A Imprinting and Associated Diseases

**Figure 1:**
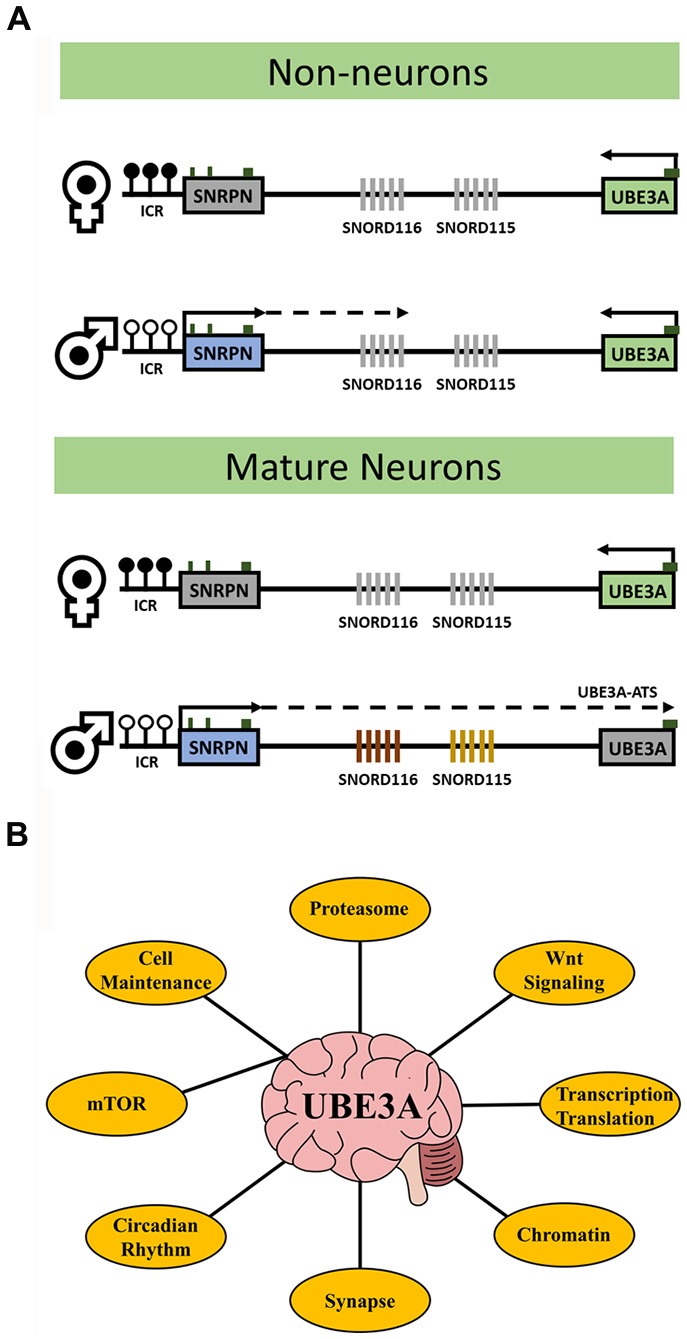
UBE3A transcriptional regulation and key related pathways. **(A)** The diagrams outline the expression and imprinting status of *UBE3A*. Maternal-specific methylation of the imprinting control region (ICR) upstream of *SNRPN* is represented by filled circles. Paternal-specific expression and transcriptional elongation through the locus results in the expression of the *UBE3A* antisense transcript (*UBE3A-ATS*) that is responsible for *UBE3A* imprinting. In neurons, paternal expression of *SNRPN* through *UBE3A* leads to transcription of the *SNORD116* and *SNORD115* clusters and induces *UBE3A* silencing *via* the *UBE3A-ATS*. In non-neurons, paternal *SNRPN* transcription does not progress to transcribe the *UBE3A-ATS* and *UBE3A* is expressed biallelically (note that non-neuronal transcription of paternal *SNRPN* stops upstream of *SNORD116* in mice but stops just downstream of *SNORD116* in human). White circles indicate unmethylated ICR, black circles indicate methylated ICR, green boxes indicate location of CpG islands, and gray fill indicates unexpressed genes. **(B)** The major processes affected by altered UBE3A levels in neurons discussed in this review. UBE3A has diverse functions and no single mechanism explains the phenotypes observed in UBE3A-related disorders. Understanding how these processes are connected *via* UBE3A my be key for therapeutic intervention.

UBE3A is an E3 ubiquitin ligase that targets proteins for proteasomal degradation. The UBE3A gene resides within the human 15q11.2-q13.3 locus that is parentally imprinted in neurons (Figure 1A) leading to the non-Mendelian inheritance patterns of three human neurodevelopmental disorders. Prader-Willi syndrome (PWS) results from 15q11.2-q13.3 paternal allele deletion whereas Angelman syndrome (AS) is caused by deletion of the maternal allele. In contrast, 15q11.2-q13.3 duplication (Dup15q) syndrome, a genetic cause of autism spectrum disorder (ASD), arises from duplications of the maternal allele. In neurons, *UBE3A* becomes silenced on the paternal allele due to the paternal-specific expression of an anti-sense transcript (*UBE3A-ATS*) originating from the unmethylated allele of *SNRPN*. *UBE3A* is the imprinted gene implicated in the maternal-specific effects of 15q11.2-q13.3 deletion or duplication disorders (LaSalle et al., 2015). However, a large population-based study recently demonstrated that paternal duplications of 15q11.2-q13.3 are associated with increased risk of ASD or developmental delay (Isles et al., 2016). How paternal transcripts, including *UBE3A* or *UBE3A-ATS*, may contribute to this finding is currently unknown.

## Evolution of Parental Imprinting of UBE3A Within Mammals

How an E3 ubiquitin ligase contributes to the pathogenesis of neurodevelopmental disease is also poorly understood. To gain functional insight into UBE3A, it is important to consider its evolutionary origin. The UBE3A gene predates the emergence of the nervous system but its imprinting was established relatively recently. After diversification of marsupials and placental mammals, multiple chromosomal rearrangements formed the domain controlling maternal expression of *UBE3A* from non-imprinted elements (Rapkins et al., 2006). Sequence data from an ancestral mammal were more similar to marsupials in chromatin arrangement suggesting that *UBE3A* imprinting evolved within mammalian radiation (Zhang et al., 2014). The ancestral non-imprinted *UBE3A* may explain its array of functions outside neurodevelopment while establishment of neuronal *UBE3A* imprinting coinciding with higher mammalian cognition may explain the link between them. The origin of UBE3A in ancient eukaryotes follows other human postsynaptic proteins that are also linked to neurogenetic disease (Bayés et al., 2011). Genomic imprinting, including that of *UBE3A*, may have evolved to regulate hibernation and sleep patterns to promote early mammalian survival at the Cretaceous–Paleogene boundary (Lovegrove et al., 2014; Tucci, 2016). The key events of *UBE3A* evolution likely included expression and localization at the synapse, colocalization with substrates essential in neurodevelopment, and acquisition of imprinting (Sato, 2017). UBE3A imprinting may have been critical for its neuronal role and understanding its establishment may pinpoint networks and pathways affected by UBE3A-associated disease.

## Cellular Localization of UBE3A and Its Alternatively Spliced Isoforms

Immunohistochemical studies show UBE3A localization to both nuclear and cytoplasmic compartments of mature neurons. A proposed role for UBE3A in transcriptional regulation is consistent with nuclear localization (Nawaz et al., 1999; Bernassola et al., 2008; Pal et al., 2013). However, many reported UBE3A substrates are cytoplasmic proteins in the ubiquitin pathway. High-resolution light and electron microscopic immunocytochemistry of UBE3A has shown a broad neuronal distribution including both axon terminals and euchromatin-rich nuclear domains (Burette et al., 2017). Additionally, localization to the mitochondria supports the notion that UBE3A regulates oxidative metabolism (Su et al., 2011; Llewellyn et al., 2015). Strong localization to axon terminals indicates physiological significance of UBE3A for the function of individual synapses whereas its nuclear localization in euchromatin-rich domains indicates a role in mediating global neuronal physiology *via* transcription regulation. This suggests that UBE3A locally regulates individual synapses while also influencing global neuronal physiology through regulation of chromatin and transcription. Furthermore, recent evidence indicates sequence variation among UBE3A’s alternatively spliced isoforms helps to determine dendritic functions (Miao et al., 2013). Further study of individual isoforms, their localization, and subsequent roles will be necessary to discriminate UBE3A’s distinct functional localizations.

UBE3A and its interaction partners appear to integrate several cellular processes including translation, intracellular trafficking, and cytoskeleton regulation necessary for neuronal functions. Those interacting proteins discussed in this review are summarized in Table 1. Proteomic analysis of UBE3A binding proteins revealed that UBE3A binds to HERC2, another E3 ubiquitin ligase, in a complex of unknown function referred to as the HUN (HERC2, UBE3A, and NEURL4) complex (Martínez-Noël et al., 2012). Network analysis of UBE3A-associated proteins, including MCM6, SUGT1, EIF3C, and ASPP2, revealed that UBE3A-associated proteins are involved in several fundamental cellular processes including translation, DNA replication, intracellular trafficking, and centrosome regulation (Martínez-Noël et al., 2018). UBE3A could be involved in the regulation of these processes either directly or as a component of the HUN complex. Interaction with MCM6 might be relevant to the transcriptional activity of UBE3A since the MCM complex interacts with RNA polymerase II and could facilitate transcription by remodeling chromatin (Yankulov et al., 1999). Binding of UBE3A to HERC2 and subsequent association with other DNA replication proteins also suggests a role of UBE3A in DNA replication and repair.

**Table 1:** Summary of notable gene interactions with UBE3A and associated pathways outlined in this review.

Gene	Type of interaction	Functions and pathways	References
HERC2	HUN complex, E3 ubiquitin ligase activity	DNA replication and repair, proteasome degradation pathway	Vos et al. (2009); Zaaroor-Regev et al. (2010); Tomaić et al. (2011) and Martínez-Noël et al. (2012)
NEURL4	HUN complex	Centriolar homeostasis	Martínez-Noël et al. (2012)
HIF1AN	Direct interaction by co-immunoprecipitation	Oxygen sensor, negative regulator of NOTCH1	Martínez-Noël et al. (2012)
MAPK6	Indirect interaction *via* HERC2	MAP kinase cascade, Ser/Thr protein kinase	Martínez-Noël et al. (2012)
MCM6	Direct interaction by affinity purification mass spectrometry	MCM complex, transcription	Martínez-Noël et al. (2012)
SUGT1	Direct interaction by affinity purification mass spectrometry	Cell cycle regulation	Martínez-Noël et al. (2012)
EIF3C	Direct interaction by affinity purification mass spectrometry	Translation initiation	Martínez-Noël et al. (2012)
ASPP2	Direct interaction by affinity purification mass spectrometry	p53 family apoptosis and cell growth	Martínez-Noël et al. (2018)
DDl1	E3 ubiquitin ligase activity	Proteasomal shuttle component	Ramirez et al. (2018)
RPN10	E3 ubiquitin ligase activity	26S proteasome regulatory subunit	Lee et al. (2014)
UCHL5	Direct ubiquitination, non-degradation	26S proteasome regulatory subunit	Lee et al. (2014)
UBXN1	Direct ubiquitination, non-degradation	ER-associated protein degradation, innate immune response	Lee et al. (2014)
CTNNB1	Direct ubiquitination, non-degradation	Wnt signaling transduction	Kuslansky et al. (2016)
EDD	E3 ubiquitin ligase activity	Proteasome degradation pathway	Vos et al. (2009), Zaaroor-Regev et al. (2010) and Tomaić et al. (2011)
PSMD4	E3 ubiquitin ligase activity	Proteasome proteolytic activity	Martínez-Noël et al. (2012) and Tomaić and Banks (2015)
BMAL1	E3 ubiquitin ligase activity	Circadian clock dynamics	Gossan et al. (2014) and Shi et al. (2015)
ALDH1A2	E3 ubiquitin ligase activity	Retinoic acid synthesis	Xu et al. (2018)
SK2	E3 ubiquitin ligase activity	small-conductance potassium channel	Sun et al. (2015b)
mTOR	Direct interaction not confirmed	Cell cycle regulation	Tang et al. (2014) and Sun et al. (2015a)
TSC2	Direct interaction not confirmed	Negative regulator of mTOR	Sun et al. (2015a)
miR-134	Ube3a1 competitive binding	miR379–410 cluster co-translation	Valluy et al. (2015)
RING1B	E3 ubiquitin ligase activity	PRC1 complex	Dunaway et al. (2016)
H2A.Z	Indirect interaction *via* PRC1	Chromatin organization, constitutive heterochromatin	Dunaway et al. (2016)

## Intracellular Pathways and Mechanistic Functions of UBE3A

UBE3A is an E3 ubiquitin ligase that poly-ubiquitinates specific intracellular proteins for degradation by the ubiquitin-proteasome system (Huang et al., 1999). Recent proteomics studies indicate that UBE3A interacts with most of the components of the proteasome, the central organelle for intracellular protein degradation (Martínez-Noël et al., 2018; Ramirez et al., 2018). A ubiquitin proteomics approach identified 13 proteasome subunits or proteasome interacting proteins, including DDl1, showed increased ubiquitination in UBE3A over-expressing *Drosophila* photoreceptor cells (Ramirez et al., 2018). DDI1 was shown to be ubiquitinated by UBE3A, without being targeted for degradation, and expressed in the developing mouse brain with a significant peak at E16.5. UBE3A also interacts with HERC2 and EDD, ubiquitin ligase components of the proteasome degradation pathway (Vos et al., 2009; Zaaroor-Regev et al., 2010; Tomaić et al., 2011). Additionally, direct interaction between UBE3A and the proteasome itself has been observed (Uchiki et al., 2009; Lee et al., 2014). Although the function of UBE3A in the proteasome is still unclear, its association with PSMD4 suggests it might help control the proteolytic activity of the proteasome. AS-associated mutants were shown to strongly interact *via* PSMD4 with the proteasome, resulting in inhibition of the proteolytic activity of the proteasome (Tomaić and Banks, 2015). These data suggest that mutant, catalytically-inactive forms of UBE3A can cause functional deficits of the proteasome. Cellular stresses that increase polyubiquitinated protein levels also blocked UBE3A from ubiquitinating the proteasome and increased proteasome activity (Jacobson et al., 2014). This suggests the proteasome can detect global polyubiquitinated protein levels and that UBE3A is involved in adjusting proteasomal activity. This perturbation of overall proteasome function may be part of AS pathogenesis.

The interaction between the proteasome and UBE3A has also been shown to induce Wnt signaling, the group of signal transduction pathways that regulate cell fate determination, cell migration, and neural patterning during embryonic development. Wnt signals regulate adult neurogenesis as well as neural stem cell behavior during central nervous system development (Kléber and Sommer, 2004; Lie et al., 2005; Kuwabara et al., 2009). Abnormal Wnt signaling is also implicated in autism pathogenesis (De Rubeis et al., 2014; Ernst, 2016; Packer, 2016). Furthermore, a *de novo* autism-linked UBE3A mutant (UBE3AT485A) prevents UBE3A catalytic inhibition by disrupting protein kinase A (PKA) phosphorylation inhibition toward itself and other substrates (Yi et al., 2015). This disruption caused enhanced UBE3A activity with increased turnover of UBE3A substrates in patient-derived cells and excessive dendritic spine development with increased synapse number in the brain. UBE3AT485A protein ubiquitinated multiple proteasome subunits leading to reduced abundance and activity, while stabilizing nuclear β-catenin and stimulating canonical Wnt signaling compared to wild-type UBE3A. This indicates that UBE3A regulates Wnt signaling and that an autism-linked mutation enhanced its signaling effects, which is corroborated by other studies that place UBE3A within the Wnt signaling pathway (Lichtig et al., 2010; Sominsky et al., 2014; Kuslansky et al., 2016). These findings also suggest that PKA helps regulate UBE3A activity during postnatal neuronal maturation to ensure proper synaptic development. This model is further supported by observations that persistent PKA inhibition does not increase dendritic spine density in *Ube3a*-deficient neurons while overexpression of UBE3AT485A profoundly increased dendritic spine density *in vivo* (Yasuda et al., 2003; Lu et al., 2011).

The role of UBE3A in regulating circadian rhythms has also emerged as an important pathway in understanding disease etiology. Ubiquitin-mediated turnover of circadian clock proteins was first observed in *Drosophila* and Neurospora (Naidoo et al., 1999; He and Liu, 2005). A link between neuronal imprinting of *UBE3A* and central clock components have been observed *via* regulation of BMAL1. UBE3A binds and degrades BMAL1 in a ubiquitin ligase-dependent manner suggesting that regulation of circadian dynamics *via* modulating BMAL1 turnover is an endogenous role of UBE3A (Gossan et al., 2014). Moreover, inactivation of UBE3A expression in AS-model mice increases BMAL1 in brain regions that control circadian behavior including enfeebled circadian activity and slowed molecular rhythms, including lengthened circadian period and reduced amplitude (Shi et al., 2015). Importantly, unsilencing the paternal allele restored functional circadian periodicity in neurons but did not alter periodicity in non-imprinted peripheral tissues. These findings constitute a mechanistic connection between circadian rhythmicity and sleep abnormalities in AS. The lengthened circadian period leads to delayed phase. This could explain why 75% of AS patients suffer from sleep disturbances, including short sleep duration and increased sleep onset latency (Smith et al., 1996; Pelc et al., 2008), one of the most stressful manifestations reported by AS families (Goldman et al., 2012).

## Synaptic Roles for UBE3A

Of interest to the understanding of UBE3A in neurodevelopment is its effect on neuronal processes and synapses. Increased UBE3A dosage was shown to negatively regulate *ALDH1A2*, the rate-limiting enzyme of retinoic acid synthesis, leading to impaired post-synaptic homeostasis (Xu et al., 2018). The loss of UBE3A in adult AS model mice results in reduced spine density in the cerebellum and hippocampus (Dindot et al., 2008). These highlight the importance of proper UBE3A dosage in synapse formation and maintenance. During the first postnatal month, elimination of dendritic spines is higher in neurons of AS compared to wild-type mice. However, spine maintenance and density were indistinguishable for mice raised in darkness, suggesting that impaired experience-driven spine maintenance leads to decreased spine density in AS model mice (Kim et al., 2016). This demonstrates that light exposure is an important environmental factor that interacts with *UBE3A* mutation to reduce dendritic spine density and disrupt cortical circuitry. How this light-dependent synaptic change in the AS mouse model may influence UBE3A’s impact on circadian factors, such as BMAL, is currently unknown.

Additionally, UBE3A has been shown to interact with small-conductance potassium channels (SKs), which are critical for learning and memory, rhythmic activity, and sleep (Adelman et al., 2012; Ohtsuki et al., 2012). UBE3A directly ubiquitinates SK2 in the C-terminal domain, facilitating endocytosis (Sun et al., 2015b). Postsynaptic SK2 levels are increased in UBE3A-deficient mice, resulting in decreased NMDA receptor activation and impairs long-term synaptic plasticity in the hippocampus. Importantly, synaptic plasticity and fear conditioned memory deficits in UBE3A-deficient mice were restored by blocking SK2. UBE3A loss in GABAergic neurons resulted in AS-like increases in neocortical EEG delta power, enhanced susceptibility to seizures, and lead to accumulation of clathrin-coated vesicles (CCV) at the presynapse without decreasing GABAergic inhibition onto pyramidal neurons (Judson et al., 2016). Conversely, UBE3A loss in glutamatergic neurons fails to show the same phenotypes, despite impairing tonic inhibition onto pyramidal neurons supporting a role of UBE3A in GABAergic neuron circuit hyperexcitability in AS mice.

Finally, UBE3A has been shown to have an important interaction with the mTOR pathway, an intracellular signaling pathway important in regulating translation, cellular metabolism, and implicated in long-term synaptic plasticity and memory (Man et al., 2003; Sui et al., 2008). Studies in ASD human brain showed dendritic spine pruning defects and impaired mTOR-autophagy that was confirmed by mTOR overactivation causing spine pruning defects in ASD mouse models (Tang et al., 2014). Furthermore, these pruning defects and ASD-like behaviors were corrected after treatment with rapamycin, an inhibitor of mTOR. Additionally, neuronal autophagy further enabled spine elimination suggesting that developmental spine pruning requires mTOR-regulated autophagy and its activation corrects synaptic pathology and social behavior deficits in ASD models (Tang et al., 2014). Furthermore, imbalanced signaling, with increased mTORC1 and decreased mTORC2 activation, leads to UBE3A deficiency-induced cerebellum-dependent motor dysfunction (Sun et al., 2015a) and hippocampal synaptic plasticity and fear-conditioning memory deficits in an AS mouse model (Sun et al., 2016). Either mTORC1 inhibition or mTORC2 activation restored long-term potentiation (LTP) and actin polymerization in AS mice hippocampus. Decreased mTORC2 activity in AS mice was reversed by rapamycin, indicating that mTORC1 over-activation leads to reduced mTORC2 activity in AS mice. Increased mTORC1 could also increase Arc levels that stimulate AMPA receptor endocytosis leading to the LTP and learning deficits seen in AS mice (Sun et al., 2017). These demonstrate the importance of mTOR balance in AS, however the specific mechanistic link between *UBE3A* and mTOR and how it contributes to AS phenotypes is not yet understood.

## Mammalian Neurodevelopment and Imprinting of UBE3A

That UBE3A has distinct localization, expression and targeting patterns during different stages of mammalian development suggests the importance of timing in intervention for treatment of UBE3A-associated disorders. Particularly in AS, determining the time at which UBE3A reinstatement is able to rescue all pertinent phenotypes, including behavioral abnormalities, cellular dysfunction, and cognitive function, will be most crucial (Sell and Margolis, 2015). Cre-dependent, neuronal induction of maternal UBE3A during developmental timepoints identified distinct windows where UBE3A re-expression can rescue phenotypes in AS mice. Maternal UBE3A induction in adolescent mice restored motor deficits, however, *in utero* reinstatement was required to rescue anxiety, repetitive behavior, and epilepsy phenotypes (Silva-Santos et al., 2015). In contrast, hippocampal synaptic plasticity could be restored in AS mice at any age. These findings indicate that therapeutic intervention early in development may be required to prevent most phenotypes associated with AS.

Another important factor in assessing UBE3A function is *UBE3A-ATS* transcribed in the opposite orientation to *UBE3A*. Transcription of *UBE3A-ATS*, or perhaps *UBE3A-ATS* itself, may introduce additional functions of both coding and non-coding UBE3A isoforms expressed in mammalian neurons. One hypothesis for why certain genes become imprinted is as a dosage-regulating mechanism. However, no correlation was found between imprinting status and expression levels of UBE3A after examination of cells and tissues among different species (Hillman et al., 2017). Alternatively, this study found that neuronal loss of paternal UBE3A protein levels during neurogenesis in mice were fully compensated by an accompanying increase in maternal UBE3A protein levels. Consistent with this finding, previous studies of mouse brain development as well as human tissues have shown UBE3A transcript level remain relatively constant (Kohama et al., 2011; Galiveti et al., 2014) and supports the emerging hypothesis that dosage compensation may not be a common reason explaining evolutionary selection of imprinted genes (Baran et al., 2015). These findings instead indicate that imprinting of UBE3A *via* the *UBE3A-ATS* may have been selected in mammals to more intricately regulate isoforms of UBE3A and not just overall expression levels.

Recently, *Ube3a1* RNA, a transcript encoding a truncated, catalytically inactive UBE3A protein, was shown to prevent dendrite growth and promote spine maturation in rat hippocampal neurons (Valluy et al., 2015). *Ube3a1* function was independent of its coding sequence and predicted to act as a long noncoding RNA (lncRNA) with a unique 3′ untranslated region containing microRNA (miRNA) binding capabilities. *Ube3a1* knockdown increased activity of miR-134, which regulates plasticity, suggesting that *Ube3a1* lncRNA acts as a competing endogenous RNA, or “RNA sponge” for miR-134. In rat neurons, *Ube3a1* transcript sequestered miRNAs from the miR379–410 cluster, which contains miR-134, thereby regulating translation of miR379–410 targets in dendrites. During development, increased neuronal activity and subsequent increased *Ube3a1* RNA levels buffered miR379–410 activity allowing progression to spine maturation (Valluy et al., 2015). These findings indicate that *Ube3a1* lncRNA may help regulate the spatiotemporal control of mRNA translation within dendrites. Many questions remain about the regulation and function of *Ube3a1* including its imprinting pattern, if the paternally expressed *UBE3A-ATS* is required for *Ube3a1* expression, and its relevance in human AS.

Finally, we have begun to explore the chromatin-related genome-wide effects of UBE3A dysregulation in human brain and neurons. We previously observed that elevated UBE3A in Dup15q syndrome had widespread effects on the neuronal methylome that converged in the dysregulation of chromatin and synaptic gene pathways (Dunaway et al., 2016). This study identified many differentially methylated genes in Dup15q compared to control brains with functions in voltage-gated ion channels, cell adhesion, signal transduction, and transcriptional regulation. Additionally, we observed a chromatin association between UBE3A and histone H2A.Z. UBE3A degrades RING1B, a known UBE3A target that monoubiquitinates histones H2A and H2A.Z, thereby regulating H2A.Z monoubiquitination. Additionally, we took a multi-layered genomics approach to identify the global effects of different UBE3A expression levels in human neuronal cell culture models revealing significant effects on DNA methylation leading to differentially methylated regions (DMRs) in genes involved in transcriptional regulation and brain development (Lopez et al., 2017). This revealed a significant effect of reduced UBE3A levels on the methylation of up to half of known imprinted genes, suggesting a role for UBE3A in a neuronal imprinted gene network. This provides strong support for a genome-wide, epigenomic function of UBE3A influencing DNA methylation and regulation of other imprinted genes in neurons.

## Summary

UBE3A has long been linked with ASD and is causal in AS etiology, however how UBE3A leads to disease phenotypes is not well understood. More recently, UBE3A genome-wide functions may enlighten additional gene pathways relevant to neurodevelopmental disorders (Table 1). Recent proteomics studies have uncovered a strong link between UBE3A and regulation of the proteasome and subsequent activation of the Wnt signaling pathway in early brain development. Aberrant UBE3A expression has large influence on proper maintenance of circadian rhythmicity and increasing evidence shows this interaction to be key in the manifestation of AS and ASD phenotypes. Synaptic functions of UBE3A including neuronal excitability may be linked to the proper balance of mTOR signaling in developing neurons. Finally, the regulatory landscape of UBE3A may also be compounded by epigenetic functions such as regulation *via* the *UBE3A-ATS* and direct influences on chromatin dynamics and genome-wide DNA methylation including regulation of other imprinted genes. Understanding the functions of UBE3A in a neurodevelopmental context will improve the study of its associated disorders and may lead to enhanced therapeutic options at key targets and pathways (Figure 1B).

## Author Contributions

SL, JL, and DS all participated in the writing and editing of the manuscript.

## Conflict of Interest Statement

The authors declare that the research was conducted in the absence of any commercial or financial relationships that could be construed as a potential conflict of interest.
